# Comparison of Intravenous Regional Anesthesia with Single-Cuff Forearm Tourniquet and Hematoma Block and Traditional Method in Patients with Distal Radius Fractures; A Randomized Clinical Trial 

**DOI:** 10.30476/BEAT.2020.46446

**Published:** 2020-04

**Authors:** Arash Farbood, Saeed Khademi, Ramin Tajvidi, Minoo Hooshangi, Saeed Salari, Mandana Ghani, Sakineh Tahmasebi, Hamid Jamali

**Affiliations:** 1 *Anesthesiology and Critical Care Research Center, Shiraz University of Medical Sciences, Shiraz, Iran*; 2 *Department of Anesthesiology, Shiraz University of Medical Sciences, Shiraz, Iran*; 3 *Shiraz University of Medical Sciences, Shiraz, Iran*; 4 *Acute pain Service nurse, Chamran Hospital, Shiraz, Iran*; 5 *CRNA, Chamran Hospital, Shiraz, Iran*

**Keywords:** Pain, Postoperative, Anesthesia, Conduction

## Abstract

**Objective:**

To investigate the effect of intravenous regional anesthesia with single-cuff forearm tourniquet and hematoma block on intraoperative and postoperative pain intensity of patients with distal radial bone fracture.

**Methods:**

In this randomized clinical trial, a total number of 52 patients with distal radius fractures were randomly assigned to receive either a traditional Bier block with 3 mg.kg^-1^ lidocaine (D group) or a single-cuff forearm tourniquet intravenous regional anesthesia with 1.5 mg.kg^-1^ lidocaine and a hematoma block with 10 mL 0.5% bupivacaine (S group). Pain intensity score of *numerical rating scale (NRS) *was measured hourly for 6 hours, then every two hours till 12^th^ hour and every 4 hours until 24^th^ postoperative hour. Total morphine consumption in the first 24 hours after surgery, its side effects and the patients’ global satisfaction were assessed in each group.

**Results:**

Mean total morphine consumption during the first 24 hours after surgery was 11.68±7.88 mg in group D and 7.12±4.42 mg in group S (*p*=0.13). Pain intensity score of NRS both during recovery room and surgical ward stay was less in S group compared to D group (0.016 and 0.02, respectively).

**Conclusion:**

Intravenous regional anesthesia with single cuff forearm tourniquet and hematoma block compared to the traditional Bier block reduced intraoperative and postoperative pain intensity more effectively in patients with distal fracture of the radius bone and also reduced morphine consumption during the first 24 hours after surgery.

## Introduction

 According to the American Society of Anesthesiologists practice guideline, over 80% of patients who undergo surgical procedures experience acute postoperative pain and around 75% of those with postoperative pain report the severity as moderate, severe, or extreme. The advantages of optimal pain management are well recognized. Nevertheless, treatment of postoperative pain keeps on being a major challenge and deficient postoperative pain relief remains disturbingly frequent [1]. Several studies have shown that despite the present-day changes in pain management, many patients still suffer from moderate to severe postoperative pain. Severe pain may result in decreased patient satisfaction, delay in postoperative ambulation, development of chronic postoperative pain, high incidence of pulmonary and cardiac complications, and increased morbidity and mortality. Distal radius fractures represent 17% of all skeletal fractures, are the most frequent fractures in young and elderly population which often occurs after falling down [1].

Studies have argued whether this kind of fracture should be treated by conservative or operative methods. Choosing a management method depends on many factors, however closed reduction and percutaneous pinning is one of the most common surgical techniques. By the guidance of radiological imaging this procedure can be accomplished either under general or regional anesthesia. Bier block, a frequently used regional technique, is effective, safe and reliable, with minimal reported complications. However, limited time of tourniquet tolerance by patients and the rapid onset of postoperative pain shortly after tourniquet deflation can be considered as disadvantages of this anesthetic technique [1]. An ideal intravenous regional anesthesia solution should have a rapid onset of action and a good potency, reduce tourniquet pain and prolong post-deflation analgesia. This may be achieved by addition of adjuncts to the local anesthetic compound. Anesthesiologists have been striving for many years to improve the efficacy and duration of regional anesthesia by injecting several drugs such as tramadol, nalbuphine, butorphanol, neostigmine and ketorolac which barely reduces postoperative pain for a long period of time [2-5]. Use of a single-cuffed tourniquet in forearm has been introduced for intravenous regional anesthesia since a few decades ago [6]. This method allows administration of lower doses of local anesthetic drugs [7]. 

In this study, we decided to investigate if this dose reduction can provide the possibility of applying a second regional block for postoperative pain relieve in patients with distal radius fracture, using this modification of intravenous regional anesthesia (Bier block with a forearm tourniquet), combined with another regional technique namely, hematoma block. The aim of this study was to determine the effect of hematoma block (which is a common method in decreasing pain of distal radius fracture reduction) combined with Bier block in intraoperative and postoperative analgesia of distal radius fracture fixation surgery. 

## Materials and Methods

*Study population*

This study was a parallel, double-blind, randomized controlled clinical trial with trial registry number of IRCT201604223213N4, in which 52 patients aged 18 to 70 years, with ASA 1 or 2 and candidates of closed reduction and percutaneous pinning of distal radius fracture were enrolled. The patients were selected from those who referred to Shahid Chamran Hospital, affiliated with Shiraz University of Medical Sciences in Shiraz, Iran. The study was approved by ethical committee of the university and written consent was obtained from participants (IR.SUMS.Med.RED.1394.91). Exclusion criteria were peripheral neuropathy (e.g. caused by diabetes), sickle cell anemia, peripheral vascular disease like Raynaud's disease, glucose-6-phosphate dehydrogenase (*G6PD*) *deficiency, history of uncontrolled convulsion, local infection or open wound in the affected upper limb, chronic use of pain-killers or opioid dependency, patient refusal, kidney or liver insufficiencies, inability to learn pain numerical rating scale or PCA pump usage, very obese or very lean patients (BMI less than 18.5 or more than 30) and history of hypersensitivity to the study drugs. *

*Randomization and intervention*

The selected patients were randomly assigned to receive one of the two interventions of group D or S by block wise randomization, 4 patients in each block, using www.randomizer.org®

*Study protocol*

Data gathering during the procedure, in recovery room and surgical ward was performed by trained nurses who were blind to the study group of each patient. The patients were also partially blind to the study group, since they were not aware of the hematoma block performed after establishment of Bier’s block. At this point, all patients were taught about how to use the patient-controlled analgesia (PCA) pump and to response numerical rating scale (NRS) pain assessment tool in which zero indicated no pain and 10 the worst pain one can imagine. After establishment of the standard monitoring devices (pulse oximetry, electrocardiography, and non-invasive blood pressure monitoring) and an intravenous line in patient’s intact hand, a distal vein at injured limb was also cannulated by a 22-gauge angiocatheter. 

*Follow-up and outcome measurement*

Pain intensity score of NRS during initial traction for reduction of radial bone was evaluated and recorded. Time to first patients complain of tourniquet pain (initiated from the inflation time) was also recorded. Any time during operation, if patient reported a pain intensity of more than 4 (according to the NRS scale), a 50 µg bolus dose of fentanyl was intravenously administered. If pain persisted, the same fentanyl doses would be repeated up to 3 µg.kg-1 or until patient’s respiratory rate dropped to less than 10 breaths/min. Unless this measure could relieve patient’s pain, general anesthesia would be induced and participant was excluded from the study citing the cause. At the end of surgery, tourniquet inflation time was recorded and the patient was transferred to the recovery room. Time to first request of patient for analgesic (beginning from tourniquet release) was measured and recorded. In recovery room, severity of pain was assessed and reported using NRS scale every 15 minutes. If the value was between 4 to 7, one mg morphine sulfate would be injected intravenously every 5 minutes, while patient’s vital signs were monitored. NRS score of 8 or more was treated by 2 mg morphine, bolus doses every 5 minute until it dropped below 8 in which case it was managed like the previous situation, until the pain intensity diminished to less than 4. 

The total dose of morphine administered in recovery room was recorded. During the recovery room stay, any signs or symptoms related to systemic toxicity of local anesthetic medication (seizure, cardiac dysrhythmia, tinnitus, perioral paresthesia, dizziness and lightheadedness) was assessed and recorded. After patient transferred to surgical ward, a PCA pump loaded with a 20 ml syringe containing 0.5 mg/mL morphine solution prepared and connected to patient’s intravenous (IV) line. The pump setup was as bolus dose of 2 mL (1 mg morphine), lockout interval of 7 minutes, 4 hours’ limitation of 60 ml (30 mg morphine) and without any baseline infusion. During first 24 hours after surgical procedure, the patient’s pain was evaluated hourly for 6 hours, then every 2 hours for next 6 hours and every 4 hours; thereafter by means of NRS. The morphine side effects (pruritus, urinary retention, nausea and vomiting and respiratory depression) were sought and recorded every 4 hours. Any time during these predetermined visits or between them, if patient experienced a pain with severity of more than 4, this was treated as mentioned for dealing pain in recovery room. The total amount of morphine consumed (the amount of drug administered through the pump plus extra doses injected by pain nurses) in the first postoperative 24 hours was calculated and recorded. Finally, overall patient’s satisfaction regarding intra- and post-operative pain control was evaluated with a 5-point scale (0=totally dissatisfied, 1=dissatisfied, 2=No comment, 3=satisfied, and 4=totally satisfied). 

*Statistical analysis*

Sample size was calculated based on the results of a pilot study in which 20 patients were randomly allocated in two limbs of the study. Analysis of the collected data showed results to be statistically significant with α=0.05 and β=0.80, and to recruit a total number of 28 patients (14 subjects in each group). To increase the power of the study and to compensate for any possible dropouts, 52 patients were included. 

To determine the normality of quantitative data, Kolmogorov-Smirnov test was used and quantitative data such as age with normal distribution were compared with Student t-test. Variables without normal distribution such as pain intensity during bone traction and reduction, the onset of tourniquet pain, the amount of fentanyl and morphine required, and the first time each patient asked for pain-killer medication were compared between the two groups with Mann-Whitney U test. Qualitative variables including sex, complication of drugs and patient’s satisfaction were analyzed by Chi-square test. ANOVA repeated measurement test was used to compare the mean pain intensity values of NRS and total amount of morphine between and within two groups and also was used to analyze the interaction between time and group. Data analysis was performed by SPSS software (version 23, Chicago, IL, USA). The significance level was considered less than 0.05. 

## Results

**Fig. 1:**
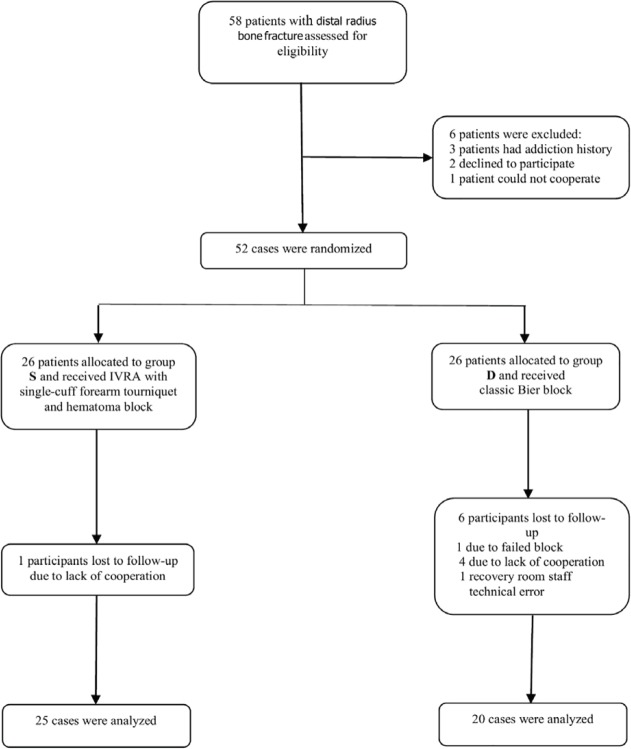
CONSORT Flowchart of the study

*The present study was performed on 52 patients with distal radius fracture scheduled for closed reduction and *percutaneous pinning. Overall, 45 patients completed the study, 20 patients in group D and 25 patients in group S (Figure 1 presents the study flowchart). Seven patients did not complete the study but their data were included in the statistical analysis till the point of their participation (intention to treat). Table 1 shows the demographic data of the studied patients. As can be seen there was no significant statistical difference between the two groups. 

Table 2 demonstrates the comparison of the data collected during the operation. Overall, 6 patients in group D and 10 patients in group S did not complain of tourniquet discomfort. At this point, one patient in group D was excluded because of failed Bier block and underwent general anesthesia. The median time to first request for pain-killer medication was 15 minutes (IQR=10-22) in group D and 30 minutes (IQR=15-105) in group S (*p*=0.002). The median value of morphine injected in recovery room was 4 mg (IQR=2-5) for group D, and 0.5 mg (IQR=0–2) in group S (*p*<0.0001). 

**Fig. 2:**
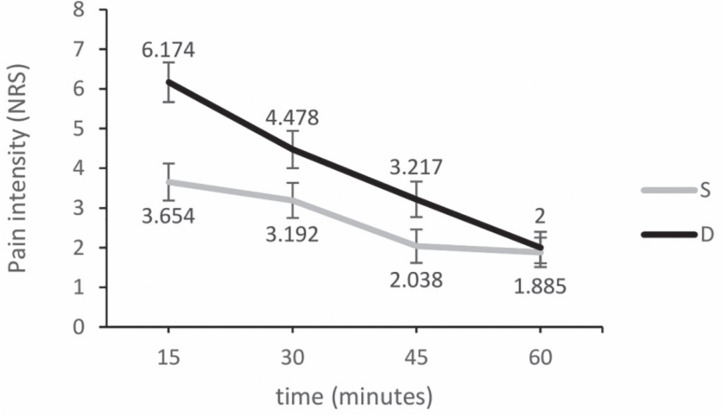
Pain intensity changes in recovering room

One patient in group D was excluded because of unawareness of recovery room staff of the study protocol, which meperidine instead of morphine was prescribed. The changes in pain intensity of NRS in recovery room have been illustrated in Figure 2. Data analysis with repeated ANOVA test indicated a statistically significant difference between the two groups at all times in favor of the S group intervention (*p*=0.016). The difference was also significant considering the effect of time (*p*=0.001) and also interaction between time and groups (*p*=0.004). 

**Fig. 3:**
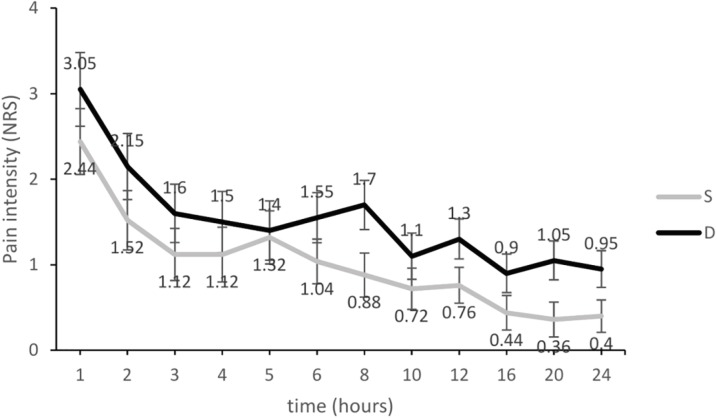
Pain intensity changes during the first postoperative 24 hours

Figure 3 demonstrates pain intensity changes of NRS in the two groups of the study after the patients were transferred to the ward and in the first postoperative 24 hours. Analysis by repeated ANOVA test showed a significant difference in measured data between the two groups (*p*=0.02). The effect of time in each group in consecutive measurements was significant (*p*=0.001). However, the interaction between time and group was not significant (*p*=0.3).

Mean values (±SD) of totally administered morphine during the first 24 hours after operation, including recovery bolus doses, the amount injected by PCA pump plus the doses administered by nurse staffs compared with independent t-test was 11.68±7.88 mg in group D and 4.7±42.12 mg in group S (*p*=0.013). Table 3 shows the patients’ global satisfaction from intervention used for pain relieve. As can be seen, there is no statistically significant difference between the two groups (*p*=0.42).

**Table 1:** Demographic data (mean ± SD) analyzed by Student-T and Chi-Square tests

**Variable**	**Group D (N=26**)	**Group S (N=26)**	**P value**
Male/Female ratio	12/14	10/16	0.575
Age (Years)	43.7213.10	42.4216.30	0.756
Weight (kg)	64.9011.27	62.769.01	0.681

**Table 2:** Data gathered during the operation (analyzed by Mann-Whitney U test)

**Variable**	**Group D**	**Group S**	***P*** ** value**
The onset of tourniquet pain from cuff inflation (minutes)^a^	20 (15-23)	25 (16-30)	0.02
Pain intensity during traction and reduction (NRS)^a^	5 (4-6)	0 (0-4)	<0.0001
The amount of fentanyl administered during surgery (g)^a^	100 (50-100)	50 (7.5-100)	0.01
Tourniquet time (minutes)^b^	43.0±10.99	40.92±10.05	0.48

All patients’ data were included till the point of their participation. ^a^Median (IQR); ^b^Mean±SD. NRS: *Numerical rating scale*

**Table 3:** Patients’ global satisfaction (analyzed by Chi-Square test)

**Group**	**Totally satisfied** **N (%)**	**Satisfied** **N (%)**	**No comment** **N (%)**	**Dissatisfied** **N (%)**	**Totally dissatisfied** **N (%)**	**P value**
D	11 (55%)	4 (20%)	5 (25%)	**-**	**-**	0.42
S	8 (32%)	13 (52%)	4 (16%)	-	-	

## Discussion

Intravenous regional anesthesia is a simple, reliable and cost-effective anesthesia technique and is widely used in forearm and hand surgeries; however, its constraints such as short duration of tourniquet tolerance by patients and the onset of postoperative pain shortly after tourniquet deflation are the disadvantages of this method [3]. In order to overcome this problem, several methods have been used so far to increase the patients’ tourniquet tolerance, as well as to reduce and delay the onset of postoperative surgical pain, mainly by administration of pain-killer compounds and drugs including tramadol [4], nalbuphine [4], butorphanol [5], neostigmine [6], and ketorolac [7] and even application of nitroglycerin skin patch [6], simultaneously with Bier block placement. 

Almost none of these compounds have reduced postoperative pain for long duration. In this study, the median time interval between tourniquet inflation and the onset of tourniquet pain, though not statistically significant, was more prolonged in S versus D group. Also, the amount of fentanyl administered during tourniquet inflation to overcome the problem was less in the S group. Chiao and colleagues in 2013 found that single-cuff forearm tourniquets would be better tolerated by the patients and required less fentanyl and propofol for their pain management [8]. In Perlas *et al.*’s study, the volunteers tolerated the rescue forearm cuff better than distal cuff of the traditional double tourniquet [9]. In the present study the time interval between cuff deflation and first request for analgesic was higher in single-cuff forearm tourniquet versus the standardized intravenous regional anesthesia group (69.4±83.58 vs 44.6±65.14 minutes). Bansal *et al*. found that using butorphanol could prolong the same time interval from 73.63±61.32 minutes in lidocaine group to 169.5±99.25 minutes in lidocaine plus butorphanol group [5].

Addition of nalbuphine to lidocaine has been shown that can prolong this time interval (211±62 vs 83±37 minutes) [10]. Abdel-ghaffar and colleagues showed that ketamine could prolong the time to first request for pain-killer medication, 20.4±3.7 hours in ketamine plus lidocaine versus 5.5±1.3 hours in lidocaine group [11]. In the present study, by measuring the pain intensity in recovery room, we found that patients experienced more severe pain at the early phase of recovery room stay, but pain intensity declined over time in both groups. Mean values of NRS were below 4 in all measurements of pain intensity in the S group. Pain intensity in the D group finally reached the mean value of the other group, but by receiving significantly more bolus doses of morphine sulfate. The same result was observed during 24 hours after surgery, it means that the pain reduction trend has continued in both groups but the patients in D group reported more severe pain than those of the S group. This shows that hematoma block added to Bier block could remarkably reduce postoperative pain in recovery room and during the first 24 hours after surgery. It has been shown that ketamine when added to lidocaine can improve postoperative analgesia during the first 24 hours [11]. Neostigmine, while showed to be ineffective in reducing postoperative pain during the first day after surgery in MacCarthney *et al.*’s research, was claimed that could relieve such pain in Sethi and Wason’s study [12, 13]. Honarmand and colleagues revealed that local (but not intravenous) ondansetron added to lidocaine, could alleviate first 24 hours’ postoperative pain after Bier block [14]. 

Although the sample size was small, there was no report of systemic toxicity of local anesthetic drugs and also that of morphine sulfate in the present study. So it seems that the major limitations of the study were first the sample size which could not address the drug complications and second, the ill-designed method of the patients’ blindness, since they could feel the location of the upper extremity tourniquets.

In conclusion, the results of the current study indicates that using hematoma block in combination with single cuff forearm tourniquet in intravenous regional anesthesia could be effectively used in closed fixation of distal radial bone fractures with more prolonged postoperative pain alleviation and less rescue pain-killer medication request. Therefore, the technique could be suggested in situations in which there is a concern about the risk of using concomitant drugs for relief of postoperative pain like obstructive sleep apnea (OSA) or hypersensitivity to sedative effects of opioids. 

## Conflict of Interest:

None declared.
